# Distance and Angle Correction System (DACS) for a kHz A-Scan Rate Pump-Probe Laser-Ultrasound Inspection

**DOI:** 10.3390/s20247266

**Published:** 2020-12-18

**Authors:** Ryan A. Canfield, Jan Ahrens, Jill Bingham, Barry Fetzer, Thomas Müller-Wirts, Matthew O’Donnell, Gary Georgeson, Ivan Pelivanov

**Affiliations:** 1 Department of Bioengineering, University of Washington, Box 355061, Seattle, WA 98195, USA; rcanfiel@uw.edu (R.A.C.); odonnel@uw.edu (M.O.); 2 TEM Messtechnik GmbH, Großer Hillen 38, D-30559 Hannover, Germany; jan.ahrens@tem-messtechnik.de (J.A.); Thomas.Mueller-Wirts@tem-messtechnik.de (T.M.-W.); 3 Boeing Research and Technology, 9725 E Marginal Way S, Tukwila, WA 98108, USA; jill.p.bingham@boeing.com (J.B.); barry.a.fetzer@boeing.com (B.F.); georgesons@msn.com (G.G.)

**Keywords:** laser ultrasound, NDT&E, automatic alignment, fiber-optic pump-probe system, laser interferometry, composite materials

## Abstract

Non-contact optical detection of ultrasound critically depends on the amount of light collected from the detection surface. Although it can be optimized in multiple ways for an ideal flat polished surface, industrial non-destructive testing and evaluation (NDT&E) usually requires optical detectors to be robust for unpolished material surfaces that are usually rough and curved. Confocal detectors provide the best light collection but must trade off sensitivity with depth of field. Specifically, detection efficiency increases with the numerical aperture (NA) of the detector, but the depth of field drops. Therefore, fast realignment of the detector focal point is critical for in-field applications. Here, we propose an optical distance and angle correction system (DACS) and demonstrate it in a kHz-rate laser-ultrasound inspection system. It incorporates a Sagnac interferometer on receive for the fast scanning of aircraft composites, which minimizes the required initial alignment. We show that DACS performs stably for different composite surfaces while providing ±2° angular and ±2 mm axial automatic correction with a maximum 100 ms realignment time.

## 1. Introduction

Non-contact interferometric methods of ultrasound (US) detection exploit either birefringence induced by US displacement in one of the interferometer arms [1,2] or record the Doppler frequency shift introduced by the surface motion due to the US wave. Comprehensive descriptions of interferometer designs applied in non-destructive testing and evaluation (NDT&E) may be found in [3,4,5]. Although interferometers can achieve high sensitivity for optically ideal (polished) surfaces under low-noise laboratory conditions, US signal reception from unpolished material surfaces in noisy industrial facilities remains a challenge.

Rough material surfaces create speckle noise and strongly reduce the light collected compared to mirror surfaces. The most efficient way to improve light collection is confocal detection. Speckle limitations can be resolved with speckle inversion using multiple photodetectors [6], photo-refractive crystals [7], or confocal Fabry-Perot [8,9] or Sagnac-type interferometers [10,11,12]. In confocal schemes, the higher the numerical aperture (NA), the larger the amount of light detected. However, the reception depth of field is inversely proportional to the NA squared. Because the probe optical wavelength is on the order of 1 μm, even relatively low (less than 0.1) NA detectors result in a sub-mm depth of field, which makes them difficult to use for in-field NDT&E applications.

One possible way to balance optimal light collection with the depth of field is to automatically control the working distance and orientation of the optical detection head relative to the sample surface for every scanning position. This can be done partially with a 5D robotic arm used for sample scanning when the 3D computer-aided design (CAD) model of the object under study is known. However, both large metallic and composite parts may have a manufacturing tolerance larger than the tolerance requirement for accurate positioning of the detection head over the part. Thus, automatic in-line self-adjustment of the probe head is still required.

Well-known applications of autofocus systems can be found in photo/video cameras, mobile phones and webcams, and CD and DVD drives for data storage. Different applications, such as NDT&E, require different approaches.

For data storage, a laser beam reads very tiny features (pits) from the surface of an optical storage medium (CD, DVD, Bluray). The pit size is on the order of the wavelength, which requires extremely sensitive focusing. Backscattered light from the media’s surface is guided to a fast photo detector, which creates digital information after complex analog and digital preprocessing. The most common auto-focusing means are the “knife edge method” [13,14] or “obscuration method” and the “astigmatic method” [15,16,17].

In the knife-edge method, the reflected beam is partially blocked by a sharp razor (knife) with subsequent analysis of power levels. In the astigmatic method, backscattered light is focused by an astigmatic lens onto a quadrant detector. Due to the different focal lengths in the X and Y directions, light is focused to a horizontal line focus at the first focal length, and to a vertical line focus at the second focal length. Depending on the lens specifications, vertical diagonal or horizontal diagonal elements are more strongly illuminated. The main advantage is very high possible measurement speed (up to tens of MHz).

Both methods are very efficient for mirror-like reflectors; however, speckle and light scattering induced by carbon fiber reinforced polymer (CFRP) surfaces makes both methods inefficient. Further challenges during surface scans are strong changes in the speckle pattern and signal intensity due to the structured CFRP surface. Today’s camera-based methods for autofocusing analyze image contrast, but composite materials often do not provide enough contrast to be highly effective. In addition, the data frame rate is usually in the order of 10 Hz, much too slow for a fast autofocus and angle stabilization system, and video processing requires a powerful digital engine. Here, we present methods to overcome these limitations for reflected light signals from highly scattering CFRP composites and show how a high-speed autofocusing and angle stabilization system can be used for rapid laser-ultrasound (LU) scanning.

An LU, kHz-rate pump-probe scanner with a fiber-optic Sagnac interferometer on receive has been presented in our previous work [10,11,13,14]. The scanner’s main components are: (i) compact, nanosecond diode-pumped laser that can lase at rates from single shot to a few kHz with about 2 mJ pulse energy to excite US signals at the surface of a target; (ii) XY translator with position-synced output that can trigger the pump laser based on position during scanning without stopping; (iii) ultra-sensitive fiber-optic Sagnac interferometer to detect echo-signals from the same sample surface.

The original fiber-optic Sagnac interferometer design was presented in [10,11,12] and then was commercialized and improved by LuxSonics Inc. It uses a low-power super-luminescent diode (SLD) but delivers a sensitivity approaching thermal noise limits. The fiber-optic Sagnac design uses the interference of optical beams reflected from the same point of a sample, one of which is shifted in time. Using polarization-maintaining (PM) fibers allows for the reception of optical fields for both interfering beams, thus making it highly insensitive to ambient noise and surface roughness. The interferometer can be compact, operate over different frequency ranges, and nearly approaches the thermal noise limit for the minimum recorded pressure [10]. These features make it nearly ideal for LU testing of CFRP composites.

A diode-pumped nanosecond laser (‘TECH Specific’, Laser-Export Ltd., Moscow, Russia) has many advantages for US signal excitation, including its high stability (both long- and short-term pulse-to-pulse energy deviations are less than 0.1%), low cost, compactness, and ability to operate at variable pulse repetition rates up to a few kHz (e.g., use it in continuous scanning mode without stopping).

The LU scanner in this study has been used for multiple NDT&E applications in composites, including flaw detection [10,11,18], ply-by-ply imaging of impact damage [19], single-sided evaluation of material porosity without requiring a back-wall signal [20], diagnostics of heat damage [21], and wrinkles [22]. Recently, it was also used to image and evaluate adhesion in glued aluminum sandwich structures [23].

The LU scanner has great potential for high-speed inspection in the field; however, positioning the detection head for complex structures is a current limitation that must be solved before this system can be widely adopted in the field. Indeed, optical detection of US at the sample surface is confocal in the fiber-optic Sagnac interferometer, and, therefore, the problem of focal spot alignment during scanning must be addressed. Here, we describe an optical distance and angle correction system (DACS) enabling fast (in less than 100 ms for complete readjustment during continuous high-speed scanning), automatic realignment of the detection spot to the optimal orientation and distance over the target surface. The distance and angular corrections in DACS are independent of each other, with operation ranges of ±2 mm and ±2°, respectively.

## 2. Methods

### 2.1. Fast Scanning Pump-Probe System

Our fast LU scanner with Sagnac interferometer on receive was described in detail and demonstrated for small composite and metal samples in previous work [10,11,18,19,20,21,22]. Briefly, the sample was fixed on an XY translator equipped with a position-based sync output (PSO). The PSO triggered the laser, producing equidistant laser firings during continuous sample translation. Note that equidistant sampling is only possible in LU if the pump laser can operate at variable pulse repetition rates because of acceleration/deceleration phases during scanning. The nanosecond laser provides pulses (about 2 mJ energy) at 1053 nm wavelength to generate US signals via thermoelastic mechanisms following absorption of the laser pulse in a thin subsurface layer of the target. Echo-signals scattered by internal heterogeneities can be recorded at the same spot (to produce B-scan and C-scan images) or at a distance from the pump (to detect surface or oblique shear waves) with the fiber-optic Sagnac interferometer powered by a tiny, low-coherent SLD source (30 mW power) at 1550 nm wavelength. Both pump and probe heads were fixed over the sample and the sample was translated in two dimensions. Note that scanning can be inverted, i.e., the sample can be fixed and a combined pump-probe head scanned by a 5D robot can be used to image large samples with complex geometries.

### 2.2. DACS Concept and Layout

**Figure 1:**
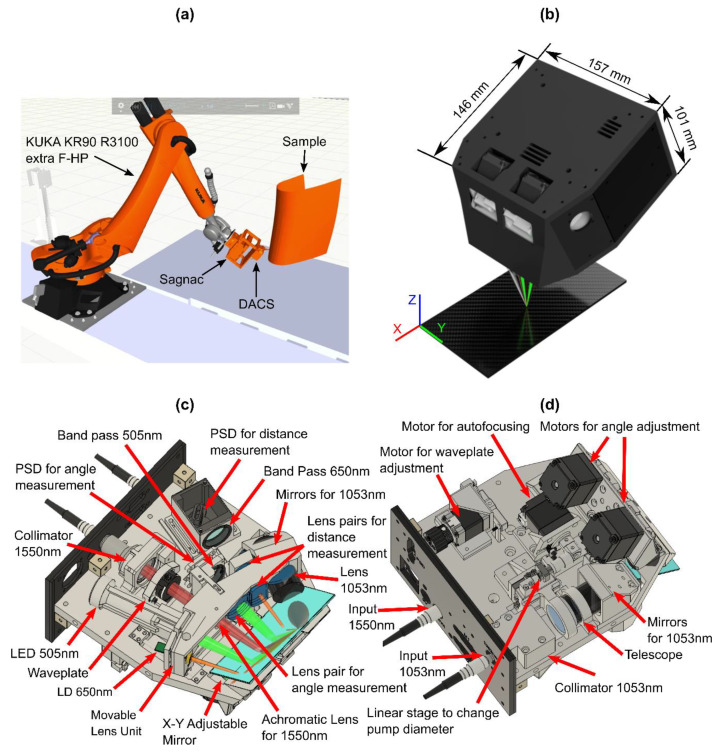
Distance and angle correction system (DACS) system integration: (**a**) future application concept, (**b**) schematic, and (**c**,**d**) component layout for a system that provides ±2° angular and ±2 mm axial automatic correction with a maximum 100 ms realignment time.

The ultimate goal of the LU system is the non-contact imaging of complicated shapes and sizes (from simple flat small samples to airplane fuselage parts). However, without detailed knowledge of target shape, accurate US imaging is nearly impossible even for contact or immersion methods. Our current approach to scanning complex samples for in-field applications is to leverage CAD models of the sample. Using these models, modern robotic arms can scan along a trajectory keeping the offset between the part and the LU detection head constant during scanning, as illustrated in Figure 1a. This approach roughly aligns the detection head with the surface. For contact or immersion US, such rough alignment is sometimes sufficient, but for the confocal LU detector with a ~1 mm depth of field, additional fine alignment is required to maximize the detection sensitivity for all scan points. Fine alignment is especially important for points where the virtual model of the sample might not exactly coincide with its factory implementation, or to compensate for the robotic arm scanning trajectory tolerance.

To implement rapid fine adjustment during robotized LU scanning, the DACS (Figure 1b–d) has been developed. We set the DACS correction ranges to be ±2 mm for the distance to the focus and ±2° for the alignment of the probe beam perpendicular to the sample surface. These ranges were chosen to match the tolerances for large aircraft components relative to the model, and the accuracy of the robot (Kuka KR90 R3100 extra F-HP) used to position the head during scanning. Generally, if the angle/distance DACS correction ranges are not enough, it is possible to feed the DACS angle and position measurement signals and regulator outputs to the robot control unit for online compensation of larger offsets during scanning. Note that we used a detection head with NA = 0.05 which gave us ~1 mm depth of field and ~ ±1° of maximum sensitivity without DACS correction.

The DACS consists of multiple components for fast opto-mechanical beam alignment (see Figure 1c,d). Its operating principle is discussed in more detail in Section 2.3. Globally, DACS combines four light sources. First, for US signal excitation, a 1053 nm wavelength pump radiation is delivered from the pulsed diode-pumped laser with a high-power multimode fiber (Thorlabs, Newton, NJ, USA) and coupled into DACS through a Sub-Miniature A (SMA) 905 connector. Second, the Sagnac interferometer is connected with DACS by a PM fiber cord (Thorlabs, Newton, NJ, USA) through a narrow key FC/PC connector to probe US echo signals from the sample surface; 30 mW low-coherent light at 1550 nm wavelength from a tiny SLD source (Thorlabs, Newton, NJ, USA) probes US signals. We used a combination of a fiber collimator and an aspheric lens mounted on a movable lens tray to focus probe radiation to the target surface. A quarter wave plate mounted on a motorized rotation stage (Figure 1c,d) optimizes polarization alignment in the Sagnac interferometer (as described previously [10]). For the measurement of the target angle and distance relative to the DACS, two more light sources (light emission diode (LED) @ 505 nm and laser diode (LD) @ 650 nm) are used in combination with position-sensitive detectors (PSD). Thus, four light sources are combined within the DACS casing.

A large aperture broadband active silver mirror (Figure 1c) delivers all light sources to the target surface and receives reflections from the alignment beams and the probe laser beam. The large mirror is directly driven by two micro stepper motors to vary the angle of the DACS output beams in the X-Y direction and keep the 1550 nm measurement beam perpendicular to the target surface.

Distance correction was performed by a movable lens tray driven by an additional micro stepper motor. The main focusing lens for the 1550 nm radiation, the LD and LED, the PSD for the angle measurement, the adjustable mirrors and focusing lens for the 1053 nm radiation and one lens pair for the distance measurement are mounted at the lens tray. To optimize US generation, the pump laser beam diameter at the target’s surface can vary from 2–4 mm by adjusting the telescope (Figure 1d).

### 2.3. DACS Operating Principle

#### 2.3.1. Distance Correction

**Figure 2:**
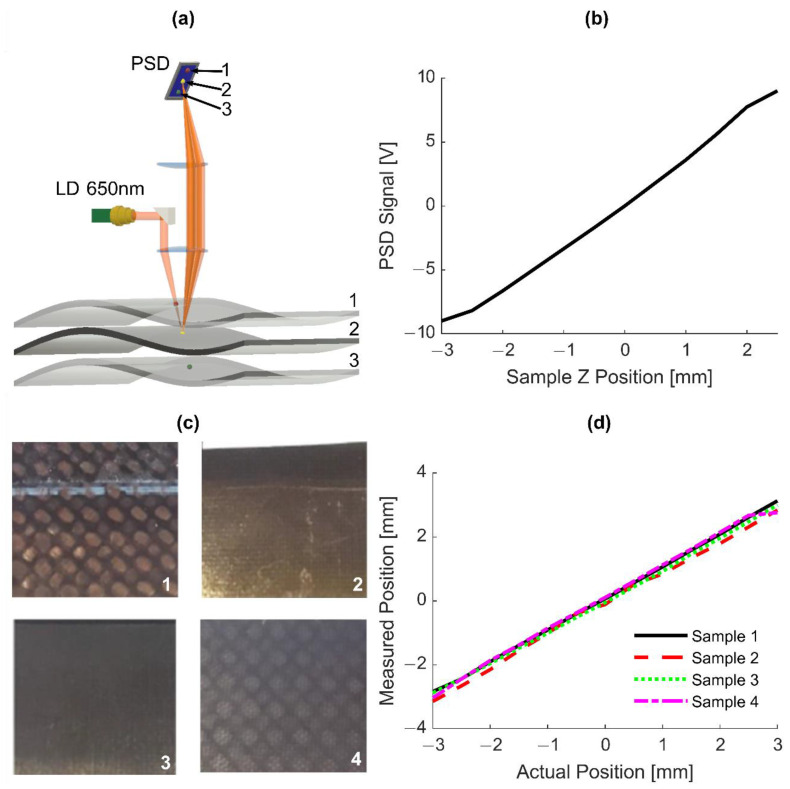
DACS operating principle for distance correction. (**a**) Sample surface position measurement concept shows how different surface levels map to different locations on a position-sensitive detector (PSD). (**b**) PSD signal output dependence on surface Z position. (**c**) Composite samples used for DACS testing. (**d**) Linearity of the measured surface position compared to the actual surface position for each sample.

The principle of distance measurement within DACS is illustrated in Figure 2a. A 650 nm ‘distance’ laser beam is focused by a lens to the target surface. This lens converts the reflected light cone into a collimated beam, parallel to the incoming beam (cat’s eye configuration). If the target is not located exactly at the focal distance, the angle of the collimated beam changes with respect to the optical axis. An additional lens transforms beam angle deviations into deviations of location of its focal spot on a PSD (see Figure 2a). Distances are optimized to get an image of the focal spot at the CFRP target surface to the PSD surface. This configuration makes detection insensitive to angular movements of the target. (Angular movement of the target produces a parallel shift of the speckle beams, but all parallel beams are focused to the same location.)

The small spot size induces large and strong speckle, especially when rough surfaces (typical for composites) are investigated. (This can also be viewed as local angle variations on the microscopic scale.) However, all speckle cones with different emission angles will be imaged to the same spot location at the PSD chip. Thus, speckle is not an issue for the proposed distance correction scheme. To reduce spherical aberrations and, therefore, improve the optical image, optimized lens pairs are used for both focusing and imaging.

For the case where the distance between the lens and target changes, the optimal PSD position for a sharp image changes as well. Using Scheimpflug’s principle [24] by tilting the PSD detector, a sharp image of the measurement laser at the target surface is achieved for different distances between focusing lens and CFRP surface.

The linearity of the detector’s voltage output based on sample Z position was investigated by mounting a piece of black paper (sample with a smooth surface structure) on the multi-axis scanner. The PSD signal dependence on target position with respect to the focus is shown in Figure 2b. Clearly, a nearly linear response is produced without any electronic corrections. This relationship was then confirmed using 4 different composite surfaces (Figure 2c,d). Given the linear behavior of the measuring signal, a reliable and robust feedback loop for distance correction can be realized. Displacement of the target surface can be compensated by shifting the focusing lens pair. The accuracy of distance correction was 200 µm on average for different unpolished surfaces of CFRP composites (Figure 2c) for the total correction range of ±2 mm. Note that a 200 µm error is still well within the depth of field of the detection system.

Within the DACS, the distance sensor is aligned for spatial overlap of the 650 nm laser and the 1550 nm laser at the target surface for the working distance with optimal 1550 nm feedback. To compensate the measured distance offset from the detection focal point, the lens was translated by a micro-stepping motor (see Figure 1c,d) controlled by a motor driver within “μAligna 144” electronics (TEM Messtechnik GmbH, Hannover, Germany).

#### 2.3.2. Angle Correction

**Figure 3:**
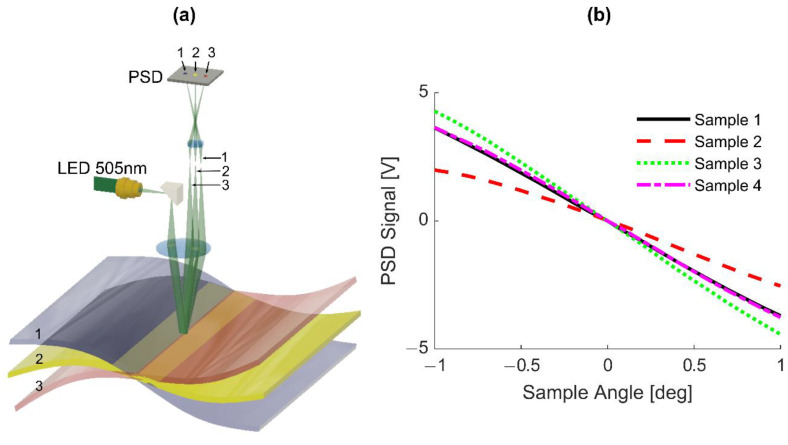
DACS operating principle for angle correction. (**a**) Sample surface angle measurement concept showing how different surface angles map to different locations on a PSD. (**b**) PSD signal output dependence on surface angle for different composite surfaces (samples correspond to their photographs in Figure 2c).

To align the probe (1550 nm) beam angularly with the surface normal, a divergent ‘angle’ measurement beam from a high-power LED (505 nm) is collimated by a lens (see Figure 3a). It creates a 5–7 mm spot size at the target surface. The spot size was far bigger than any structure on the CFRP surface to average out surface roughness. Angular movement of the reflected light beam is translated into parallel movement of a focused beam after passing through the lens. This corresponds to far-field detection. A parallel shift of the target in any direction, especially in the z-direction (axial direction), does not influence the spot location. It is solely defined by the angle of reflected light. To reduce the influence of speckle, a low coherent LED was used.

The PSD was positioned in a way that movement of the target in the z-direction produces minimum change of the beam position at the PSD. Detector performance was investigated for the same four graphite-epoxy composite samples (see Figure 2c). As shown in Figure 3b, sample angular misalignment is nearly linearly related to the PSD output over a range greater than one degree. In exchanging samples (see Figure 2c), we noticed that the angle calibration changed due to the different back-scattering characteristics of the four samples. We notice that the calibration of the angle sensor is different for each sample. However, this is not a problem if the working point is at the zero angle position of the PSD and angle stabilization is active. To stabilize at a different angle, a target dependent calibration is required. Such calibration can be easily obtained by monitoring the angle measurement signal for a defined angle movement of the mirror between the focusing lens and the target for each material type.

To compensate for angles within the target range of ±2°, a robust micro stepper motor driven actuator was developed. To reach a high regulation speed, the motors are directly connected via lever arms to the mirror. The angle correction actuator was controlled by a motor driver within μAligna 144 electronics (TEM Messtechnik GmbH, Hannover, Germany). This enables rather fast movements over the range of ±2 mm, producing mirror tilts of about ±3° in x- and y-directions.

### 2.4. DACS Assembly

**Figure 4:**
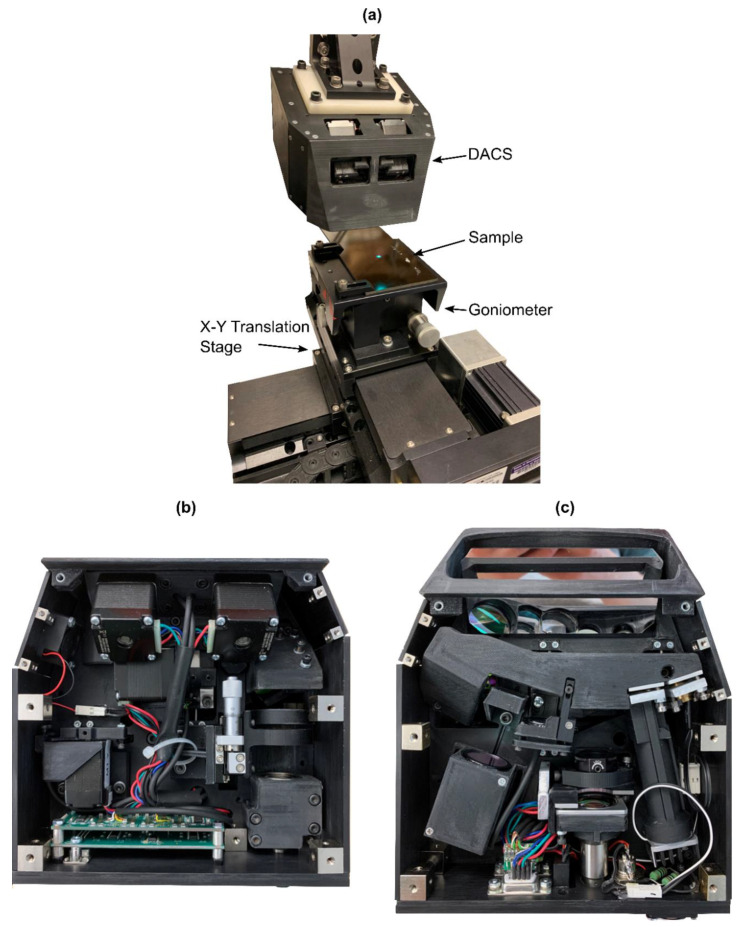
(**a**) DACS experimental setup. (**b**) DACS internal components corresponding to Figure 1d. (**c**) DACS internal components corresponding to Figure 1c.

Based on the approach described in Section 2.1, Section 2.2 and Section 2.3, the DACS unit was assembled (see Figure 4) and tested. Voltage signals from distance and angle PSD detectors were monitored during testing. At first, motor parameters (speed, acceleration, drive current, etc.) were optimized for highest speed and best performance in feed forward operation. Motor offsets were changed periodically between two set values, while corresponding detector signals were recorded with an oscilloscope. These measurements showed that the autofocus unit can compensate a 4 mm offset in less than 100 ms, while the fast mirror actuator can change angle by several degrees in less than 20 ms.

After optimizing motor parameters in forward operation, the regulator was switched to stabilize distance and angle to a defined setpoint. It was altered as distance and angle signals from the DACS were recorded. The regulator speed was optimized to achieve the fastest possible control without significant overshoot. Due to mechanical limitations (hysteresis, friction, inertia) the optimal regulator gain depends on travel distance. The final settings were chosen so that the regulator did not oscillate for compensations within the target regulator range.

Clearly, both distance and angle corrections are fast enough to perform in real-time during LU scanning and are stable for different composite surfaces typically used in industry.

## 3. Results

To evaluate the practical limitations of DACS regulation, we conducted a series of experiments assessing the allowable angular misalignment, compensation time for sample discontinuities, and performance in response to multidimensional misalignments. All experiments used a sample from Boeing with known defects, whose surface characteristics corresponded to Sample 3 in Figure 2c. Different defects were embedded about halfway through its depth. The same sample was scanned in previous publications [11,18] where the detailed arrangement of defects can be found.

The sample was placed on a XY translator (Aerotech, PRO115, Aerotech, Pittsburgh, PA, USA) with a goniometer (Aerotech, ATT185-5, Aerotech, Pittsburgh, PA, USA) on top to manually tilt the sample by a defined angle relative to either X or Y axes. The DACS reference position was initially determined by aligning the detector’s focal point.

**Figure 5:**
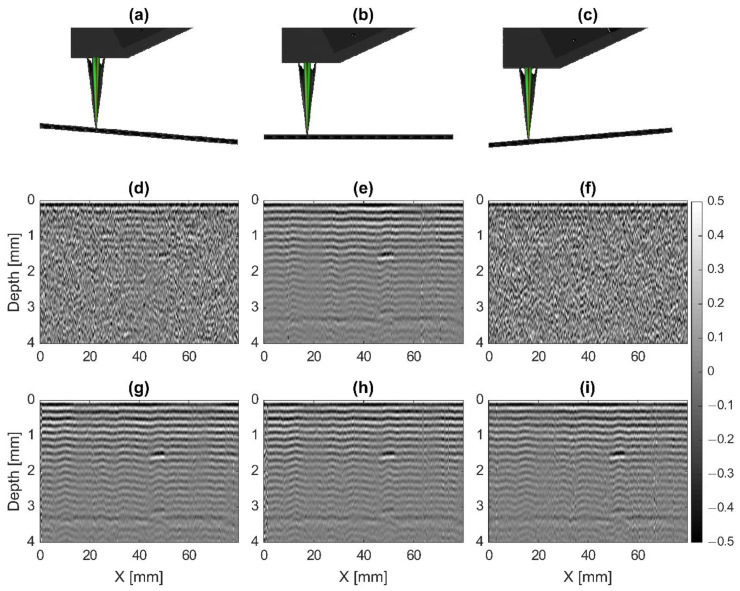
B-scan diagrams and results when the sample is rotated around the *Y*-axis with DACS regulation ON and OFF. Diagrams showing the sample rotated −2° (**a**), 0° (**b**) and +2° (**c**). (**d**–**f**) Results obtained with regulation OFF for sample positions (**a**–**c**), respectively. (**g**–**i**) Results obtained with regulation ON for sample positions (**a**–**c**), respectively.

Figure 5 shows results from manually rotating the sample about the *Y*-axis with DACS regulation OFF (Figure 5d–f) and ON (Figure 5g–i). The sample was translated 80 mm at a speed of 100 mm/s with a laser (1053 nm) pulse repetition rate of 1 kHz for each test, resulting in an X step size of 0.1 mm. This means that the pulse laser is triggered every 0.1 mm, but the scanning system and DACS operate otherwise independently. The DACS continuously auto-aligns regardless of the scanning state. A schematic of sample angular misalignment of −2° and +2° from the reference position can be seen in Figure 5a,c, respectively. When the sample is aligned with the reference position, the angular misalignment is at 0° (Figure 5b).

Figure 5e represents a typical image obtained with the LU scanner. Signal processing routines for A-, B- and C-scans were described in detail in our previous papers [11,18,19]. The very broad system bandwidth not only can image defects, but also the regular structure of composites. Note that if the regular structure is not of interest, it can be removed as described in [11] to better visualize large defects. Here, we keep the full structure image because it is a great indicator of imaging system performance and alignment.

With regulation OFF, the resulting B-scan after processing shows dramatically lower signal-to-noise ratio (SNR) when the sample is at either a −2° or +2° angle (Figure 5d,f) compared to the 0° angle case (Figure 5e). The known defect at 1.5 mm depth and 47.5 mm in the X direction are not immediately obvious in the B-Scans when the sample is misaligned. However, when DACS regulation is turned ON, the SNR at the −2° or +2° angles (Figure 5g,i) significantly improves, and matches the 0° results. In all sample orientations with regulation ON, the known defect is very clear. Notably, this experiment also tests the performance of the DACS distance measurement and compensation system. At a 2° angle, the edges of the sample are a minimum of 1.4 mm from the optimal position in the Z direction. Clearly, the DACS provides significant improvement in SNR and overall image quality over this operating range.

**Figure 6:**
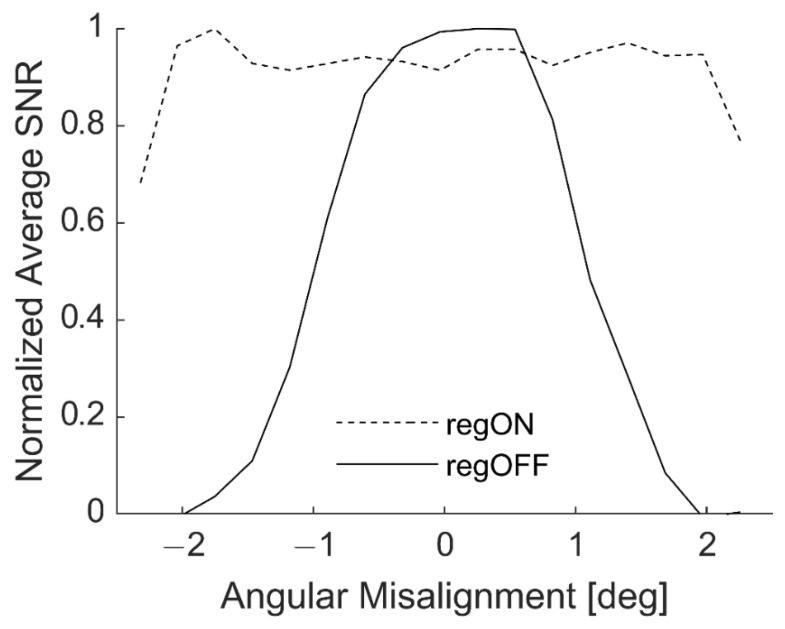
Normalized average signal-to-noise ratio (SNR) across a B-scan at different angular misalignments with regulation ON (regON) and regulation OFF (regOFF).

The next set of experiments focused on quantifying DACS performance across its entire distance and angle operating ranges. Figure 6 shows the SNR at angular misalignments in 0.25° steps from the reference position, centered at 0°, with the sample angled about the *Y*-axis. Within the design range of ±2°, the average SNR across the B-scan is nearly constant with DACS regulation ON. While the average SNR peaks at 0° with DACS regulation OFF, it tapers off smoothly as the sample approaches ±2° angular misalignment. When DACS regulation is turned ON, the SNR is driven close to the optimal focal position, but is prone to small fluctuations because the DACS regulation continuously adjusts the current focal point of the system based on the position and alignment of the sample surface. These fluctuations prevent the DACS, with regulation ON, from achieving the physically optimal performance achieved at 0° misalignment with regulation OFF. Future updates to the DACS controller could pause continuous regulation within a certain range of angular misalignment near 0° and achieve the physically optimal SNR. At ±2°, the mirror actuator reaches its mechanical limit while the direction of misalignment is well known. This information can be easily used to rotate the DACS head manually (or with the robot arm) and bring the actuator back within its range to increase the possible angle stabilization range.

**Figure 7:**
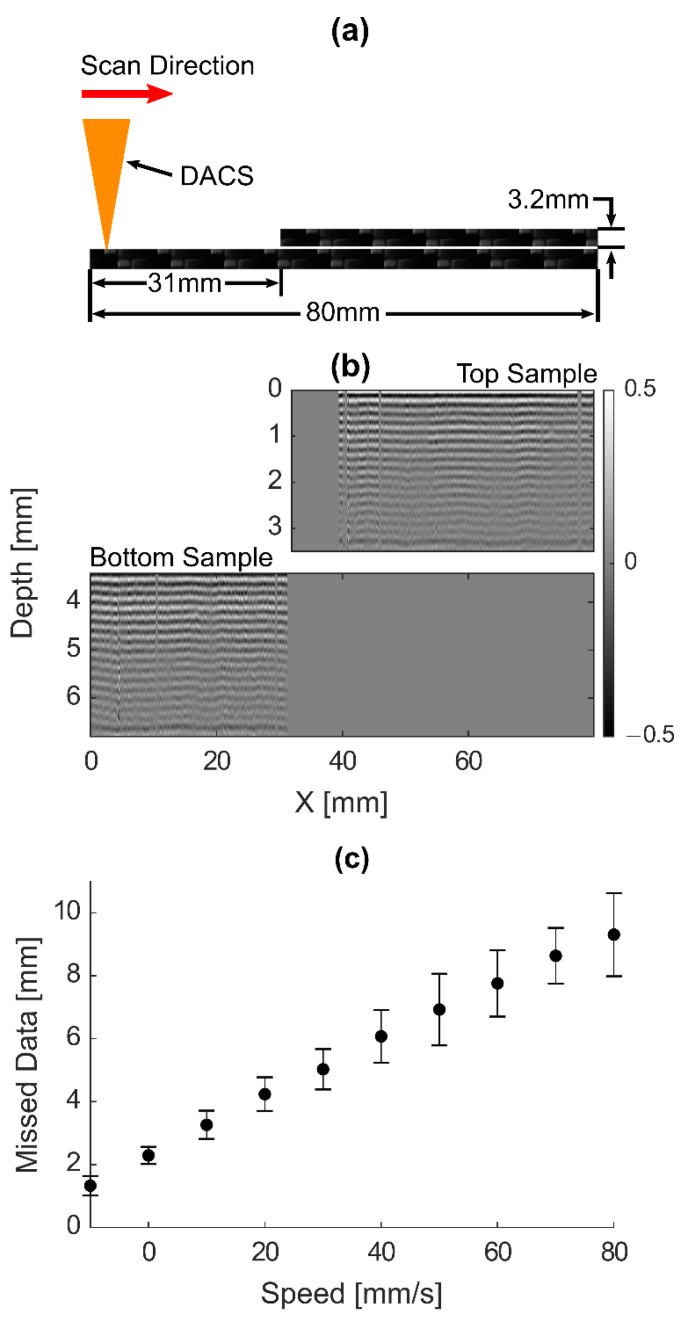
(**a**) Schematic of the experimental scan setup with two 3.2 mm thick samples on top of each other. (**b**) B-scan performed from left to right with DACS regulation ON through an interface where the second composite sample was placed on top of the first with no acoustic coupling to the first sample. A 9 mm dead zone (a zone with missing data) is evident for a scanning speed of 100 mm/s, which corresponds to a response time of 90 ms. (**c**) Length of missed data for B-scans performed at different scan speeds in response to a 3.2 mm step in the Z direction of the sample resulting from a roughly 100 ms response time for all scan speeds.

Another important practical characteristic of DACS is its response to discontinuities. When the distance PSD detects an abrupt change in surface position, the DACS requires some time to mechanically adjust the mirror and autofocus to optimize optical alignment at the new surface position. To determine its response time, two staggered and overlapping samples were placed within the scan range. The DACS was initially aligned on the surface of the bottom sample and a scan was performed over both samples, as illustrated in Figure 7a,b, which presents a B-scan with the scan direction from left to right. It clearly shows roughly a 9 mm area of missed data from the top sample after the discontinuity, resulting in a response time from the DACS of less than 0.1 s when scanning at 100 mm/s.

Multiple different speeds in 10 mm/s intervals between 10 and 100 mm/s were then analyzed, and the consistency of a 0.1 s response time from the DACS was obtained for all scanning speeds. As can be seen in Figure 7c, the response time is nearly 0.1 s regardless of speed, although the variance of response time increases with speed as well.

**Figure 8:**
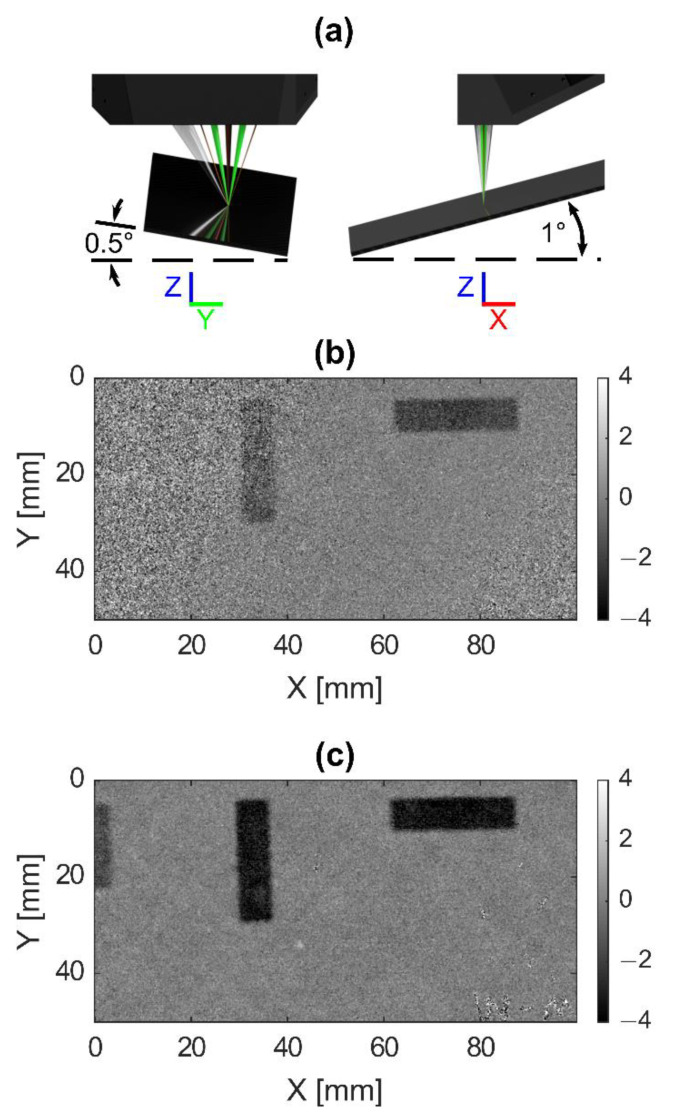
(**a**) Diagram of sample tilt 1° about the *Y*-axis, and 0.5° about the *X* axis for two-dimensional laser-ultrasound (LU) scanning. C-scans at a 1.5 mm sample depth for DACs regulation OFF (**b**) and ON (**c**).

Lastly, the sample was tilted 1° about the *Y*-axis and 0.5° about the *X* axis (see Figure 8a) before performing a 100 by 50 mm scan. The translation rate was 100 mm/s, with an X and Y step size of 0.1 mm, and a laser (1053 nm) pulse repetition rate of 1 kHz. US data were low pass filtered with a cutoff frequency of 10 MHz prior to image reconstruction to remove the structure signal so that known defects can be easily visualized. With DACS regulation OFF, the defects can be partially recognized as the darker regions in the C-scans of Figure 8b; however, the results are noisy. In contrast, with DACS regulation ON, these known defects can be clearly seen in Figure 8c. In particular, the defect ranging from 0 to 4 mm in the X direction and 5 to 22 mm in the Y direction cannot be seen with regulation OFF but is clearly visible with DACS regulation ON.

## 4. Discussion and Conclusions

Although confocal optical detection trades off sensitivity with depth of field, we have shown here that the limited depth of field of a tightly focused detection beam can be compensated with a proper feedback system. In other words, if the detector focal point moves out of alignment with the surface, the distance and angle correction system, which we call DACS, will automatically realign the detector to the position of maximum sensitivity.

We designed DACS to improve probe light collection for LU scanning in NDE applications. It combines pulsed pump (1053 nm) radiation and continuous probe (1550 nm) low-coherent light so that the maximum sensitivity point of probe light is in the center of the pump beam at the sample surface. To stabilize the detector focal point during scanning, angle and distance corrections were implemented using additional 650 nm LD and 505 nm LED sources, respectively. Thus, DACS contains four different light sources (pump, probe and two applied for distance/angle alignment). Fiber-optic components used in the Sagnac interferometer transmit light in a very narrow wavelength range centered at 1550 nm. Thus, radiation from additional sources at any other wavelengths will not be coupled into the interferometer and, therefore, will not alter the detection characteristics.

The principle advantage of the DACS design is that the distance from the probe light focal point and the angle from the sample normal are aligned independently. Surface movement up and down does not affect angle alignment, and out-of-plane surface rotation does not affect distance alignment. This enables the unambiguous decoding of light positions on the PSDs, for both angle and distance alignment beams, converting them to inputs for the fast translators controlling the rotation mirror and autofocus system. The total time for full angular and distance stabilization was measured to be less than 100 ms.

LU scanning with DACS is more robust for complex geometries, extending the effective detector depth of field from ~1 mm to 4 mm and stabilizing detection to a ±2° rotation relative to the sample normal. The absolute magnitude of the stabilization range is not large compared to variations in typical part geometries, especially when the shape of the sample under investigation is unknown. However, we intentionally reduced the DACS working range to make the correction stable, unambiguous, and fast.

Confocal LU detection with an autofocus adjustment module does not provide benefits in alignment over cumbersome Doppler or cavity-based detectors [25,26] (which have much lower sensitivity and tremendously higher probe light power but much more flexible in the detector’s misalignment) for scanning quasi-flat panels. For parts with complicated geometry requiring robotized scanning around the target, however, the benefits in using DACS are quite clear.

As we mentioned in the introduction, DACS was never intended as a stand-alone tool for LU scanning of arbitrary parts with unknown geometries. The target application is robotic scanning of large composite parts where the geometry is well known and described in CAD models (see Figure 1a). In particular, DACS is intended to compensate for small deviations from the virtual model of the sample and inaccuracies in the scanning trajectory, i.e., for fine adjustment. Fine adjustments on-the-fly enable high-speed scanning of complex parts with an LU system, thus bringing the high image quality of LU to field applications of NDE. The specific choice of angle and distance operating ranges was specified by the scanning needs at Boeing. An additional application is similar to what we showed in the Results section; that is, for LU scanning of a relatively small sample to remove image artifacts related to slight sample curvature and to greatly simplify alignment before scanning.

In general, DACS parameters such as working distance and operating range can be adjusted for specific NDE needs. For example, internal DACS signals (target angle and distance and DACS regulator output) can be sent to the robot’s controller, which will optimize the position and orientation of the DACS head by the scanning robot to increase the alignment range by an order of magnitude. Because DACS operation is fully independent of the pump and probe beams, it can be combined with any other LU systems where probe beam alignment is critical.

The geometry presented in Figure 7 illustrates a potential limitation caused by DACS finite response time (~100 ms). If there is a sharp surface transition, then there is a partial loss of data after the transition, e.g., blind zones are created in the image. At high scan rates, the feedback system cannot follow the rapid change in surface relief. However, the scan speed near sharp surface changes can be locally adjusted. This can be done automatically using the regulator signals, which clearly show if the regulator is locked or not. As mentioned above, the pump nanosecond laser supports variable repetition rates and, therefore, can exactly follow the Trig In signal from the translator. This is a very important advantage of the LU scanner used here compared to previous LU systems utilizing bulky, high energy solid state lasers that must operate at a fixed rate. In addition, blind zones appear after sharp transitions and, if the scan is repeated in the opposite direction, they will appear on the opposite side of the transition. Combining two scans should completely remove these blind zones.

Our future work will focus on integrating the DACS-stabilized LU scanner on a robotic arm to demonstrate the efficient and high-quality inspection of large aircraft components with complex geometries.
